# Applications of Chitin and Its Derivatives in Biological Medicine

**DOI:** 10.3390/ijms11125152

**Published:** 2010-12-15

**Authors:** Bae Keun Park, Moon-Moo Kim

**Affiliations:** 1 Institute of Basic Medical Science, Yonsei University Wonju College of Medicine, Wonju 220-701, Korea; E-Mail: baekpark@yonsei.ac.kr; 2 Department of Chemistry, Dong-Eui University, Busan 614-714, Korea

**Keywords:** chitin, chitosan, antioxidant, anticancer, anti-inflammatory, drug delivery

## Abstract

Chitin and its derivatives—as a potential resource as well as multiple functional substrates—have generated attractive interest in various fields such as biomedical, pharmaceutical, food and environmental industries, since the first isolation of chitin in 1811. Moreover, chitosan and its chitooligosaccharides (COS) are degraded products of chitin through enzymatic and acidic hydrolysis processes; and COS, in particular, is well suited for potential biological application, due to the biocompatibility and nontoxic nature of chitosan. In this review, we investigate the current bioactivities of chitin derivatives, which are all correlated with their biomedical properties. Several new and cutting edge insights here may provide a molecular basis for the mechanism of chitin, and hence may aid its use for medical and pharmaceutical applications.

## 1. Introduction

Since chitin (C_8_H_13_O_5_N)_n_ was first isolated and characterized from mushrooms, the earliest known polysaccharide, by French chemist Henri Braconnot in 1811 [1], it has been discovered to be the second most abundant natural biopolymer in the world [2–4], amounting in marine biomass alone to approximately 106–107 tons. Chitin is a long-chain homopolymer of *N*-acetyl-d-glucosamine (GlcNAc), (1–4)-linked 2-acetamido-2-deoxy-β-d-glucan, a derivative of glucose. Strong acids can split the chitin, water-insoluble polymer, into acetic acid and chitosan, and chitin can be processed further into two main derivatives of chitosan and amino glucose through many nonspecific enzymes, such as cellulases, lipases, proteases and chitosanases [5,6]. Notably, chitosan is a nontoxic biopolymer produced by the deacetylation of chitin, and currently chitosan and its oligosaccharides have received considerable attention due to their biological activities and properties in commercial applications. During the last few decades, as a source of bioactive material, chitosan and chitooligosaccharides, which are degradation products of chitin or chitosan produced by enzymatic or acidic hydrolysis, were introduced into a variety of biomedicals including wound dressings and drug delivery systems [7,8], and the food and chemical industries [9–12]. Chitin and its derivatives have delivered biological potential for a wide range of applications such as in the food and medical field [13–16], agriculture [17] and aquaculture [18,19], dental [20,21] and cosmetics [22,23], wastewater [24,25] and membranes [26,27] .

This review aims to analyze the most recent advances in biological applications of chitin and its derivatives, particularly those related to anti-inflammatory and antioxidant activities, antimicrobial effects, immunity-enhancing as well as antitumor effects and drug delivery, in the field of biological medicine.

## 2. Antioxidant Activity

Hydrogen peroxide, hydroxyl radical and superoxide anion called reactive oxygen species (ROS) [28] are generated during normal metabolism and oxidize biomolecules, such as lipids, proteins, carbohydrates and DNA, ultimately leading to oxidative stress. However, cells produce antioxidants such as catalase, superoxide dismutase, glutathione peroxidase and thioredoxins as a part of the cellular defense system against ROS-mediated cellular injury [28]. When the cellular defense mechanisms are unable to deal with excessive generation of ROS, this oxidative stress has been reported to induce various pathogenic processes including aging, cancer, wrinkle formation, rheumatoid arthritis and inflammation [29–33]. Therefore, antioxidants including vitamin C and E play an important role in maintaining balance between the oxidative and reductive state inside the body. Among these antioxidants, chitins—widely distributed among invertebrates and crustaceans as structural material in their exoskeletons and fungal cell walls—have shown important biological antioxidant effect that has potentials for a wide variety of applications [34]. However, chitin is an insoluble polymer in water, which is the major limiting factor for its utilization in living systems. Therefore, it is important to produce soluble chitin or chitosan by hydrolysis. In recent years, two kinds of chitin oligosaccharides or *N*-acetyl chito-oligosaccharides (NA-COSs) with different molecular weights [35] produced from crab chitin hydrolysis was evaluated in live cells. Two kinds of NA-COSs with molecular weights (M.W.) of 1–3 kDa (NA-COS 1–3 kDa) and below 1 kDa (NA - COS < 1 kDa) exhibited an inhibitory effect against DNA and protein oxidation. In addition, intracellular glutathione (GSH) level and direct intracellular radical scavenging effect were increased in the presence of them in mouse macrophages (RAW 264.7) and exerted an inhibitory effect against cellular oxidative stress. In particular, NA-COS 1–3 kDa was more effective than NA-COS < 1 kDa in protein oxidation and production of intracellular free radicals in live cells [13]. Furthermore, it was reported that chitin oligosaccharides (NA-COS; M.W. 229.21–593.12 Da) produced by acidic hydrolysis of crab chitin exert the cellular antioxidant effects. In addition, the inhibitory effect of NA-COS on myeloperoxidase (MPO) activity in human myeloid cells (HL-60) and oxidation of DNA and protein were identified in mouse macrophages. Moreover, their direct radical scavenging effect of intracellular hydrogen peroxide and intracellular glutathione (GSH) level were demonstrated, suggesting that NA-COS act as potent antioxidants in live cells [3]. Previous study also showed how disease resistance and ROS metabolism in harvested navel oranges are affected by chitosan. Its treatment effectively not only enhanced the activities of peroxidase and superoxide dismutase, and levels of glutathione and hydrogen peroxide, but also inhibited the activity of catalase and the decrease of ascorbate content during navel orange fruit storage, suggesting that chitosan treatment could induce navel orange fruit disease resistance by regulating the H_2_O_2_ levels, antioxidant enzyme and ascorbate– glutathione cycle [36]. Furthermore, it was demonstrated that COS smaller than 1 kDa suppressed the generation of intracellular radical species in B16F1, a murine melanoma cell line, suggesting prevention of oxidative stress related disease. In addition, induction of intracellular glutathione (GSH) level was increased in the presence of COS, which exhibited a protective effect on oxidative damage of genomic DNA independent of molecular weight [37]. Chitosan also has an *in vivo* stimulatory effect on both nitric oxide production and modulates peroxide production [38]. The protective effects of COS against hydrogen peroxide-induced oxidative damage were also evaluated in human umbilical vein endothelial cells (HUVEC, ECV304 cells). In addition to a marked decrease in intracellular ROS level, COS also exerted preventive effects on the production of lipid peroxidation such as malondialdehyde, restoring activities of endogenous antioxidants including superoxide dismutase and glutathione peroxidase, along with the capacity of increasing levels of nitric oxide and nitric oxide synthase. It was also demonstrated that COS can effectively protect HUVEC against oxidative stress by hydrogen peroxide, which might be of importance in the treatment of cardiovascular diseases [39]. In addition, COSs (M.W.1500, DD. 90%) protect against Cu(II) induced neurotoxicity in primary cortical neurons by interfering with an increase in intracellular ROS [40]. Moreover, COS of four different molecular weight ranges (below 1 kDa, 1–3 kDa, 3–5 kDa and 5–10 kDa) were investigated for their ability to protect pancreatic-β cells from damage induced by hydrogen peroxide. COS (500 μg/ml) with molecular weights of 3–5 kDa raised the viability of hydrogen peroxide-treated cells by 58.46% compared with the hydrogen peroxide alone group [41]. It was also described that COS is able to protect hydrogen peroxide-induced oxidative stress on human embryonic hepatocytes (L02 cells), suggesting that COS might be useful in a clinical setting during the treatment of oxidative stress related liver damage [42]. Administration of chitosans with low molecular weight was reported to inhibit neutrophil activation and oxidation of serum albumin commonly observed in patients undergoing hemodialysis, resulting in reduction of oxidative stress associated with uremia [43]. On the other hand, the cellular antioxidant effects of carboxylated chitooligosaccharides (CCOS), a chemically modified derivative of COS, were evaluated by the ability to inhibit lipid and protein oxidation. Radical-mediated oxidation of cell membrane lipids and proteins was inhibited by CCOS that reduced the level of lipid hydroperoxides and carbonyl carbon content in mouse macrophages. Further, CCOS inhibited myeloperoxidase activity in human myeloid cells, suggesting the indirect possibility of inhibiting generation of ROS such as superoxide radicals, H_2_O_2_ and HOCl [44].

## 3. Anti-inflammatory Effects

Although a number of studies have widely investigated the effects of chitin, chitosan and their derivatives, few investigating anti-inflammatory activity have recently been published. Inflammation is a physiological body immune response against pathogens, toxic chemicals or physical injury. While acute inflammation is a short-term normal response that usually causes tissue repair by recruitment of leukocytes to the damaged region, chronic inflammation is a long-term pathological response involving induction of own tissue damage by matrix metalloproteinases (MMPs) [45,46]. It is generally well known that chronic inflammation is related to periodontal disease, hepatitis, arthritis, gastritis and colitis. The most important factor in chronic inflammation has been known to be the nuclear factor-kappa B (NF-κB) transcription factor that plays a critical role in regulating genes involved in immune responses [47]. NF-κB is known to regulate inflammatory genes encoding pro-inflammatory cytokines, adhesion molecules, cyclooxygenase-2 (COX-2) and inducible nitric oxide synthase (iNOS) [48,49]. In particular, current approaches to the treatment of inflammation rely on the selective inhibition of COX-2 activity responsible for producing prostanoids, not COX-1. Non-steroidal anti-inflammatory drugs (NSAIDs) are the most widely prescribed drug for treatment of many inflammatory diseases. However, they display a high incidence of gastric, renal and hepatic side effects. In recent years, it has been reported that chronic inflammation is associated with an increased risk of malignant transformation [50]. This is because phagocytic leukocytes in chronic inflammatory processes produce large amounts of reactive metabolites of oxygen and nitrogen that induce oxidative stress and lead to oxidation of fatty acids and proteins in cell membrane, thus impairing their normal function. Although the anti-inflammatory effects of chitin and its derivatives have been rarely reported, in recent years data has been accumulating. First of all, it was found that chitin is a size-dependent regulator of inflammation [51]. In this study, while both intermediate-sized chitin and small chitin stimulates TNF production in murine peritoneal macrophages, large chitin fragments are inert, Furthermore, it was found that chitin stimulates the expression of TLR2, dectin-1, the mannose receptor and inflammatory cytokines, differentially activated NF-κB and spleen tyrosine kinase. Ngo *et al.* (2009) demonstrated that chitin oligosaccharides can inhibit myeloperoxidase activity in human myeloid cells and oxidation of DNA and protein in mouse macrophages [13]. Chitosan was confirmed to partially inhibit the secretion of both IL-8 and TNF-α from mast cells, demonstrating that water-soluble chitosan has the potential to reduce the allergic inflammatory response [52]. Since mast cells are necessary for allergic reactions and have been implicated in a number of neuroinflammatory diseases, chitosan nutraceuticals may help to prevent or alleviate some of these complications. In another study, it was demonstrated that chitooligosaccharides enhanced migration of the mouse peritoneal macrophages into inflammatory areas [53]. LPS-stimulated TNF-α and IL-6 secretion was found to be inhibited in the presence of chitosan oligosaccharide in RAW 264.7 cells [54], suggesting that chitosan oligosaccharide may possess an anti-inflammatory effect via the inhibition of TNF-α in the LPS-stimulated inflammation. These functions of chitosan to exert anti-inflammatory effect could be unilized in the nutraceutical industry as well as in functional foods for prevention and alleviation of inflammatory diseases. In addition, it was reported that chitosan promotes phagocytosis and production of osteopontin and leukotriene B by polymorphonuclear leukocytes, production of interleukin-1, transforming growth factor b1 and platelet-derived growth factor by macrophages, and production of interleukin-8 by fibroblasts, enhancing immune responses [55]. The effect of chitin, chitosan and their derivatives on MMPs related to chronic inflammation is an interesting topic presently. MMPs are a family of secreted or transmembrane endopeptidases that degrade extracellular matrix components. It was described that chitooligosaccharides inhibit activation and expression of MMP-2 in primary human dermal fibroblasts [56]. In particular, hydrolysed chitosans with molecular weights as low as 3–5 kDa displayed the highest inhibitory effect on MMP-2. Moreover, the inhibitory effect might be described by the effective chelating capacity of chitosan for Zn^2+^ as a cofactor of MMP-2. Based on these findings, as a result of elucidation of the relationship between their activities and structures, atomic force microscopy demonstrated a direct molecular interaction between MMP-2 and chitosan. Affinity chromatography revealed a high binding-specificity of MMP-2 to chitosan, and a colorimetric assay suggested a non-competitive inhibition of MMP-2 by chitosan [57].

## 4. Antimicrobial Effects

Chitosan and its derivatives have widespread applications in agriculture, medicine, environment, food, *etc*. The antimicrobial activity of chitin, chitosan and their derivatives against different groups of microorganisms, such as bacteria, yeast and fungi has received considerable attention in recent years. In particular, chitosan can also be used as an antimicrobial film to cover fresh fruits and vegetables [58]. Chitosan, which is a deacetylated form of chitin, has been investigated for numerous antimicrobial activities. In particular, water-soluble chitosan derivatives were evaluated for their antimicrobial activity [59]. The actual mechanism of antimicrobial activity of chitosan and its derivatives is not yet fully understood but has been suggested to involve cell lysis, breakdown of the cytoplasmic membrane barrier and the chelation of trace metal cations by the chitosan [60–64]. In the killing of gram-negative bacteria, a cationic chitosan must interact with both bacterial cell envelope membranes. The antibacterial effect of chitosan is higher than that of chitin because chitosan possesses a number of polycationic amines which can interact with the negatively charged residues of carbohydrates, lipids and proteins located on the cell surface of bacteria, which subsequently inhibit the growth of bacteria. In addition, because of the positive charge on the C-2 of the glucosamine monomer at pH values below 6, chitosan is more soluble and has a better antimicrobial activity than chitin [65]. The antibacterial effects of COS have been known to be dependent on their degree of polymerization or molecular weight. In addition, the water-soluble chitooligosaccharides may be advantageous as antibacterial agents in *in vivo* systems compared to water-insoluble chitosan. These variations in preparation methods are likely to result in differences in the deacetylation degree, the distribution of acetyl groups, the chain length and the conformational structure of chitosan [66] and will thereby have an influence on the solubility, the antimicrobial activity and other properties. The antibacterial effect of three kinds of COS with relatively higher molecular weight (HMWCOS), medium molecular weight (MMWCOS), and lower molecular weight (LMWCOS), respectively, was evaluated against various microorganisms. The molecular weight of COS is critical for microorganism inhibition and required higher than 10,000 Da. Generally, the COS have more effective activity against pathogens than nonpathogens, except in the case of lactic acid bacteria [67]. In addition to chitosan, several chitosan derivatives such as acid-free-water soluble chitosan [68] and quaternary N-alkyl chitosan [69] were reported to exert antimicrobial effect. The antimicrobial activity of chitosan depends on several factors such as the kind of chitosan (deacetylation degree or molecular weight), the pH of the medium, the temperature, the presence of several food components and so forth. The mechanism of the antimicrobial activity has not been fully elucidated yet, but several hypotheses have been postulated. The most feasible hypothesis is a change in cell permeability due to interactions between the positive charges of chitosan and the negative charges on the cell surface. This interaction between positively charged chitosan molecules and negatively charged microbial cell membranes results in the leakage of intracellular constituents. In addition, it can be suggested that chitosan and its derivatives not only bind to bacterial genes but also chelate metals such as calcium, magnesium and zinc ions, leading to the inhibition of transcription and translation. Therefore, an antibacterial mechanism of chitosan and its derivatives can be described to be exerted by above mechanisms.

## 5. Immuno-Stimulating and Anticancer Effects

**Figure 1.**
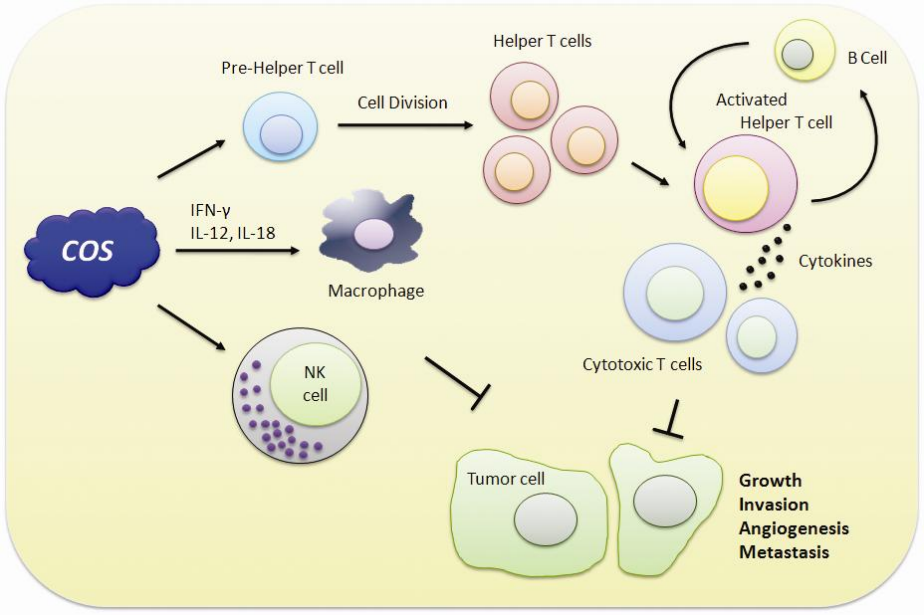
Chitooligosaccharides (COS) can display anti-tumorigenic activity through immunomodulation.

In recent years, it was revealed that the tumor inhibitory effect of COS is probably related to their induction of lymphocyte cytokines through increasing T-cell proliferation (Figure 1). Basically, the antitumor mechanism of COS is enhanced by acquired immunity via accelerating T-cell differentiation to increase cytotoxicity and maintain T-cell activity [70]. Maeda and Kimura examined the antitumor effects of various low-molecular weight chitosans, such as water-soluble 21- or 46-kDa molecules with low viscosity, produced by enzymatic hydrolysis of over 650-kDa chitosan, which displayed decreased tumor growth and final tumor weight in sarcoma 180-bearing mice due to increase of natural killer cell activity [71,72]. The results indicate the low-molecular-weight water-soluble chitosans and oligochitosans might be useful in preventing tumor growth, partly through enhancing cytotoxic activity against tumors as an immunomodulator [73]. In transdermal delivery of baicalin for an useful drug for the treatment of skin disease, low-molecular-weight chitosans can improve its permeation through mouse skin [74]. In addition, they seem to activate macrophages via the production of cytokines such as interferon (IFN)-γ, interleukin (IL)-12 or -18 from the intraepithelial lymphocytes. In examination of the anticancer activity of chitosan derivatives, there was no clear information describing the relationship between charge properties and their observed activities. In recent years, in several cell lines, one research group observed that cancer-cell viability was significantly reduced regardless of the positive or negative charge of differently charged COS derivatives [75]. Moreover, COS significantly inhibited human hepatocellular (HepG2) carcinoma cell proliferation and down-regulated cell cycle-related gene expressions with decreased DNA content and up-regulation of p21 *in vitro*. In *in vivo* observations, COS inhibited tumor growth of HepG2 and Lewis lung carcinoma xenografts and lung tumor nodules as well as lung metastasis [76]. Quan *et al*. suggest that COS act as inhibitors of heparanase, which is a β-endoglucuronidase, and assist tumor invasion, metastasis and angiogenesis [77].

Other chemically modified structures, aminoderivatized COSs, such as aminoethyl-, dimethyl aminoethyl- and diethyl aminoethyl-COS, not only significantly induce cell death but also inhibit proliferation of human gastric adenocarcinoma cells [78]. This report shows that water-soluble aminoderivatized COS might be able to serve as valuable cancer chemopreventive agents. COS could also suppress tumor angiogenesis *in vivo* and *in vitro* [79,80] through blocking migration of endothelial cells induced by nitric oxide [81,82].

## 6. Application in Drug Delivery System

To provide anticancer chemotherapy, chitosan is attracting increasing attention as drug and gene carriers due to its excellent biocompatibility, biodegradability, and nontoxicity [83,84]. Chitosan has an important role in delivery of drugs, with the potential to improve drug absorption and stabilize drug components to increase drug targeting. In addition, as a potential gene deliverer, chitosan can protect DNA and increase the expression period of genes. Chitin or chitosan derivatives, which were conjugated with some kinds of anticancer agents, can execute better anticancer effects with a decrease of side effects and gradual release of free drug in the cancer tissues. Furthermore, chitosan nanoparticles were synthesized and applied for *in vivo* antitumor activity [85–87]. On the other hand, for ocular drug delivery, liposomes coated with low-molecular weight chitosan may be potentially applicable to clinic uses [88].

Nanoparticles enable chitosan to elicit dose-dependent tumor-weight inhibition with highly impressive antitumor efficacy *in vivo* [88]. The doses and particle quantum size have a great effect on their efficacy as drug carriers. In particular, with small particle size and positive surface charge, the complex could exhibit higher antitumor activity than other chitosan derivatives [89]. Smaller sized particles seem to enhance efficacy of the particle-based drug delivery systems [90–92]. Basically, chitosan nanoparticles are produced with a mean particle size ranging from 40 to 100 nm and a positive surface charge of about 50 mV [93,94]. To introduce these products into *in vitro* cell culture systems, they should be filtered by a membrane with diameter of 0.45 μm and autoclaved [95]. In *in vivo* animal models, different administration routes of chitosan nanoparticles, such as intravenous (i.v.) or intraperitoneal injection (i.p.) and oral administration (p.o.), could exhibit little difference in antitumor activities. However, because nanoparticulate systems have been developed to improve the blood circulating time and tumor targeting efficacy of vincristine, administration of chitosan nanoparticles i.v. can contribute *in vivo* efficacy to antitumor activities [96,97] followed by a prolonged blood half-life of drugs [98].

## 7. Conclusion

In recent years, chitin and its derivatives—as a high potential resource as well as multiple functional substrates—have generated attractive interest in various fields such as biomedical, pharmaceutical, food and environmental industries. While chitin is an insoluble polymer in water, which is the major limiting factor for its utilization in living systems, COS are more suited to draw attention for potentially biological applications due to the biocompatibility and nontoxic nature of chitosan. They exert an excellent antioxidant effect as well as antimicrobial effect. In particular, COS and their derivatives are potential candidates capable of preventing or treating diverse chronic inflammation such as colitis, periodontal disease, hepatitis and gastritis, leading to cancer, and through drug delivery system.
